# Regulation of Ubiquitin Family Signaling in Disease

**DOI:** 10.3390/ijms24076735

**Published:** 2023-04-04

**Authors:** Jose Luis Rosa

**Affiliations:** Departament de Ciències Fisiològiques, Universitat de Barcelona, IDIBELL, L’Hospitalet de Llobregat, 08907 Barcelona, Spain; joseluisrosa@ub.edu

Ubiquitin is a small regulatory protein found in all eukaryotic cells. The addition of ubiquitin to a substrate protein is called ubiquitylation. This process involves a synchronized cascade of three enzyme classes: ubiquitin-activating enzyme (E1), ubiquitin-conjugating enzyme (E2), and ubiquitin ligase (E3). The result of this sequential cascade is to bind ubiquitin to lysine residues on the protein substrate via a peptide bond, to cysteine residues through a thioester bond, to serine and threonine residues through an ester bond, or to the amino group of the protein’s N-terminus via a peptide bond. Ubiquitin can be attached as a single molecule (monoubiquitylation) or in the form of polymeric chains (polyubiquitylation) with different topologies. Notably, proteins can also undergo modification by ubiquitin-like molecules such as SUMO or NEDD8. Additionally, other modifications of ubiquitin, including acetylation and phosphorylation, have been identified. These diverse modifications create a code-like system, where each modification leads to distinct outcomes within cells. Protein ubiquitylation can be reversed by deubiquitylating enzymes, which catalyze the removal of ubiquitin or ubiquitin-like molecules from the modified substrate through a process called deubiquitylation [1,2,3].

Ubiquitylation plays a significant role in regulating cellular functions, including protein degradation via the proteasome or the autophagy/lysosome pathway. Consequently, this post-translational modification is involved in nearly all aspects of eukaryotic biology, including cell proliferation, cell differentiation, DNA damage repair, cell cycle, apoptosis, signal transduction, and vesicle traffic, among others. The dysregulation of the ubiquitylation process has been associated with various diseases such as neurodegeneration, cancer, viral infections, and inflammation. Understanding the molecular mechanisms involved in all these processes and their regulation is crucial to identify potential targets for the therapeutic treatment of associated diseases [1,2,3].

The Special Issue entitled “Regulation of Ubiquitin Family Signaling in Disease” of the International Journal of Molecular Sciences includes a total de seven contributions (three original articles and four reviews) providing new information in the field of ubiquitin family regulation and its association with diseases.

Sánchez-Bellver et al. [4] discuss the important role of the ubiquitin–proteasome system in the differentiation and ciliogenesis of photoreceptor cells. Mutations in the genes involved in ubiquitination and proteostasis can cause inherited retinal dystrophies (IRD) and ciliopathies. The article focuses on USP48, a deubiquitylating enzyme, and its role in the retina. The authors found that USP48 localizes to the basal body in retinal cells and is involved in the regulation and stabilization of key ciliary proteins for photoreceptor function. USP48 also interacts with IRD-associated proteins ARL3 and UNC119a and stabilizes their protein levels using different mechanisms, indicating its importance in intracellular protein transport and ciliary trafficking to the photoreceptor outer segment.

Cho et al. [5] describe how UDP-glucose acts as a protein degrader for glucokinase, which is essential in producing glucose-6-phosphate in the pancreas and liver and is the primary target for glucose-induced insulin secretion in β-cells. The authors found that both glucose and UDP-glucose bind to glucokinase, while uridine and UDP-glucose bind to cereblon. This led to the identification of UDP-glucose as the molecular glue that links cereblon and glucokinase. The administration of UDP-glucose to β-cell lines and mice resulted in the ubiquitylation and degradation of glucokinase, leading to a reduction in insulin secretion. The authors also found that the glucokinase^E256K^ mutant protein, found in Maturity-onset diabetes of the young type 2 (MODY2) patients, was resistant to UDP-glucose-induced ubiquitylation and degradation, suggesting that the glucokinase ubiquitylation and degradation signaling pathway may be impaired in MODY2 patients.

Ghilarducci et al. [6] identify RNF167 as a novel ubiquitin ligase for Rab7, a GTPase that plays a significant role in regulating late endosome and lysosome trafficking. In vitro assays demonstrate that Rab7 is a direct substrate of RNF167 and that RNF167 activity maintains Rab7’s membrane localization. Moreover, the authors found that the GTP-bound active form of Rab7 is necessary for membrane anchoring and accessibility to RNF167-mediated ubiquitin attachment. In relation to diseases, they also demonstrate that the RNF167-mediated ubiquitylation of Rab7 GTPase is impaired by variants of Charcot–Marie–Tooth Type 2B disease.

Sala-Gaston et al. [7] discuss the role of Large HERC ubiquitin ligases (HERC1 and HERC2) in diseases. The authors highlight the importance of understanding how cell signaling is affected in these pathologies to identify potential therapeutic targets. The article reviews recent advancements in the understanding of how Large HERCs regulate the MAPK signaling pathways and suggests possible therapeutic strategies, including the use of inhibitors and proteolysis-targeting chimeras, to address MAPK signaling abnormalities resulting from Large HERC deficiencies.

Kitamura [8] summarizes recent studies about the regulator role of ubiquitin-specific proteases (USPs) in metabolic disorders. The author discusses USPs involved in hyperglycemia regulation and the progression of diabetic nephropathy, neuropathy, and retinopathy. He also highlights the current understanding of the modulatory roles of USPs in hepatic disorders such as non-alcoholic fatty liver disease (NAFLD), atherosclerosis, and Cushing syndrome.

Xu et al. [9] focus on the neuronally expressed developmentally downregulated 4 (NEDD4) subfamily, a subclass of E3 ubiquitin ligases. The authors provide a comprehensive understanding of its specific functions, downstream substrates, and upstream regulatory mechanisms in osteogenesis. Additionally, they also discuss the participation of E3 ubiquitin ligases and deubiquitylating enzymes in the development, repair, and regeneration of teeth.

Lara-Ureña et al. [10] discuss the importance of SUMOylation in cancer. The SUMO pathway is regulated by various enzymes, such as proteases and ligases, that control the sumoylation of specific targets. The dysregulation of these enzymes can result in different types of cancer. The SENP and PIAS families are among the most extensively studied proteases and ligases in this pathway. The ligases may have additional functions, making it challenging to study their SUMO-associated role in cancer development. The authors provide an up-to-date overview of the impact of the dysregulation of SUMO proteases and ligases on cancer initiation and progression.
